# An exploratory exome-wide machine learning analysis identifies candidate host gene signatures associated with Long COVID in a large admixed Brazilian cohort

**DOI:** 10.3389/fmed.2026.1837186

**Published:** 2026-07-24

**Authors:** Aléxia Stefani Siqueira Zetum, Danielle Ribeiro Campos da Silva, Vinícius Do Prado Ventorim, Felipe Ataides Mion, Felipe dos Santos Passarela, Henrique Perini Rosa, Túlio de Lima Campos, Bartolomeu Acioli-Santos, Flávio Rosendo da Silva Oliveira, Karen Ruth Michio Barbosa, Livia Cesar Morais, Raquel Silva dos Reis Trabach, Lorena Souza Castro Altoé, Yasmin Moreto Guaitolini, Patrícia Brasil, Matheus Correia Casotti, Iúri Drumond Louro, Débora Dummer Meira

**Affiliations:** 1 Department of Science Biology, Federal University of Espírito Santo, Aracruz, Espírito Santo, Brazil; 2 Hemostasis Laboratory, Hemotherapy and Hematology Center of Espírito Santo, Vitória, Espírito Santo, Brazil; 3 Bioinformatics Core Facility, Oswaldo Cruz Foundation, Recife, Pernambuco, Brazil; 4 Department of Virology, Aggeu Magalhães Institute, Oswaldo Cruz Foundation, Recife, Pernambuco, Brazil; 5 Federal Institute of Education, Science and Technology of Pernambuco, Recife, Pernambuco, Brazil; 6 Oswaldo Cruz Foundation, Rio de Janeiro, Brazil

**Keywords:** genetic risk, Long COVID, neuroinflammation, polygenic interaction, XGBoost

## Abstract

**Introduction:**

Genetic factors have been suggested as modifiers of vulnerability to postCOVID-19 sequelae, referred to as Long COVID (LC). We hypothesize that LC may involve central nervous system (CNS)-related mechanisms, influenced by neuroinflammatory, autoimmune, viral mechanisms, and genetic factors. In this work, we used whole-exome sequencing in conjunction with a machine learning-based prioritization framework to investigate the connection between LC and host genomic variation.

**Methods:**

Our patient group included 312 individuals previously infected with SARS-CoV-2 enrolled in two public hospitals of Vitoria city, Brazil, between November 2020 and July 2023. After rigorous quality control in accordance with reference guidelines, the exome data revealed 651,652 variants in our cohort. To rank candidate variants, a supervised machine learning framework combining Recursive Feature Elimination (RFE) and XGBoost was implemented. Five variants were found to be statistically significant after Benjamini–Hochberg false discovery rate (FDR) correction in subsequent logistic regression analyses that were adjusted for age, sex, and principal components of ancestry under an additive genetic model.

**Results:**

The variant at *LERFS* - rs200443822 was associated with increased odds of LC (OR = 6.21, 95% CI 2.98–12.91, FDR-adjusted *p* < 0.01). Similarly, the variant at *PNKD* - rs1870125 (OR = 2.33, 95% CI 1.52–3.57, FDR-adjusted *p* < 0.01) and *LIPA* - rs1051338 (OR = 2.87, 95% CI 1.77–4.65, FDR-adjusted *p* < 0.01) showed increased odds. In contrast, variants at chromosome 10 (rs7912524 - *HK1* and *LAMB4* - rs1735499) were associated with reduced odds of LC, with ORs ranging from 0.38 to 0.50 (all FDR-adjusted *p* < 0.01).

**Discussion:**

Our findings do not support single-gene causal effects; rather, they are consistent with the notion that common variants may collaboratively influence inter-individual variations in LC manifestations within a more extensive polygenic framework. Clinical features such as fatigue, pain, anosmia, dysautonomia, and cognitive impairment may reflect interactions between host genomic background and clinical or demographic factors. Overall, this study provides a hypothesis-generating integrative framework for investigating host genetic contributions to LC in an underrepresented admixed population. Targeted functional studies are important to ascertain the biological significance and translational applicability of these findings.

## 1 Introduction

COVID-19, initially understood as a purely respiratory infection, is now defined as a multisystemic disease with comprehensive and long-lasting clinical effects (1, 2), affecting millions of people worldwide.

With the increase in reports of persistent symptoms, the World Health Organization (WHO) defined this condition as Long COVID (LC) and highlighted the need for new clinical approaches to understanding the disease. This condition is defined by manifestations that last at least 2 months, appearing up to 3 months after infection and not explained by other diagnoses (3).

The pathophysiology of LC remains poorly understood. Proposed mechanisms include viral persistence in tissues, prolonged immune dysregulation, reactivation of latent viruses (EBV, HHV-6), molecular mimicry, autoimmunity, alterations in gut microbiota, and microvascular thrombosis with endothelial dysfunction (4).

LC presents a complex array of symptoms, with fatigue, cognitive impairment, and sleep disturbances among the most frequent and disabling. Notably, such manifestations have also been observed in vaccinated individuals and in mild cases of COVID-19 (5). Given that 10 to 30% of infected individuals may develop long-term sequelae, understanding and addressing these persistent symptoms is a critical public health priority. Evidence from previous coronavirus outbreaks, such as SARS-CoV-1 and MERS-CoV, already indicated a risk of post-viral neuroinflammation (6). In the case of SARS-CoV-2, recent studies point to the involvement of the Central Nervous System (CNS), including a higher incidence of neurological and psychiatric disorders in the months following infection (7). This has been confirmed through neuroimaging of post-COVID-19 patients and by the identification of structural and functional brain alterations (8). These findings support the notion that LC may be linked to a CNS-related condition resulting from viral, autoimmune, or neuroinflammatory mechanisms (9).

Therefore, this study sought to explore possible relationships between host genetic variation and CL in a mixed-race Brazilian cohort. Through whole-exome sequencing and an exploratory prioritization framework based on machine learning (ML), we selected a set of variants that may be involved in the observed clinical heterogeneity. The results will be presented and discussed in subsequent sections.

## 2 Materials and methods

### 2.1 Ethics declarations

This research was approved by the Human Research Ethics Committee of the Health Sciences Center/UFES, under protocol number CAAE: 37094020.6.0000.5060. It is a retrospective cohort study for exome sequencing in patients who developed COVID-19. All volunteers personally signed an Informed Consent Form (ICF) authorizing the performance of molecular tests. All experiments were conducted in accordance with relevant guidelines and regulations.

### 2.2 Patients and control group

There were 491 people in our cohort (Supplementary Table S1). Of these, 226 were recruited from two reference hospitals in the Greater Vitória metropolitan area, Espírito Santo, Brazil: Hospital Estadual Dr. Jayme Santos Neves (HEJSN) and Hospital Unimed Vitória – HU (CIAS), from November 2020 to December 2021 (10). In addition, 86 biological samples were obtained from the State Center for Hemotherapy and Hematology Dr. Marcos Daniel Santos (HEMOES). Another 179 participants were enrolled through institutional partnerships, including the Physical Therapy Clinic of the School of Health Sciences of Santa Casa de Misericórdia de Vitória (EMESCAM), the Physical Rehabilitation Center of Espírito Santo (CREFES), the Cassiano Antônio Moraes University Hospital (HUCAM), FIOCRUZ Rondônia, and FIOCRUZ Rio de Janeiro. Social media platforms were also used to actively recruit volunteers. All the volunteers gave an ICF and completed a clinical-epidemiological questionnaire prepared as per WHO guidelines for COVID-19 and LC (11). Acute COVID-19 severity was classified according to WHO criteria and based on clinical records from the participating hospitals as: (i) mild disease, symptomatic cases without supplemental oxygen requirement; (ii) moderate/severe disease, hospitalization requiring supplemental oxygen; and (iii) critical disease, intensive care unit (ICU) admission and/or mechanical ventilation. SARS-CoV-2 infection was confirmed at the time of the acute episode by either real-time reverse transcription polymerase chain reaction (RT-PCR) or rapid antigen testing, according to the diagnostic protocols adopted by the participating public hospitals. Among 491 persons recruited, 397 had their genetic materials assessed. But for the final analyses, only 312 persons who were not vaccinated against SARS-CoV-2 prior to infection development were included (206 with LC and 106 without LC). Whole blood samples were kept refrigerated (2–8 °C) until the DNA extraction step.

### 2.3 Genomic DNA extraction

The QIAamp Mini Kit (QIAGEN^®^, Hilden, Germany) was used to process the samples, and the NanoDrop^®^ (Thermo Scientific, United States) and the Qubit^®^ (Life Technologies, United States) were used to measure the concentration and purity of the DNA. 6.67 ng/μL was the final concentration used for sequencing.

### 2.4 DNA library preparation and genotyping

Whole-exome sequencing was performed at the National Institute of Cardiology (INC) following the Illumina DNA Prep with Enrichment protocol (San Diego, CA). Quantification was carried out using the Qubit dsDNA BR Assay Kit, while fragment size and distribution were assessed with the Agilent Bioanalyzer (Santa Clara, CA).

### 2.5 Data preprocessing

For Data preprocessing, the Illumina DRAGEN software was used (San Diego, CA). The methodology employed was Small Variant Caller: Germline, utilizing the reference genome hg38-altmaskedv3-graph-enabled and the Illumina Exome Panel v1.2 (CEX) to delineate target regions. The Nirvana software was used to add notes to the variants that were found. After sequencing, the FASTq, Binary Alignment/Map (BAM), Index File (BAI), and Variant Call Format (VCF) files were saved on Illumina’s BaseSpace Sequence Hub platform.

### 2.6 Quality and variant filtering

The quality of the VCF files generated by DRAGEN was evaluated using DISCVRSeq (v1.3.62) (12). Variant filtering was performed with VCFtools (v0.1.16) (13) according to predefined thresholds, including minor allele frequency (MAF ≥ 0.05), minimum variant quality score (Phred ≥20), minimum read depth (DP ≥ 5), and a maximum of 10% missing genotypes per variant. Hardy–Weinberg equilibrium testing was applied exclusively to the control group (*p* > 1 × 10^−5^ threshold), and variants deviating from equilibrium were excluded. Variant-level quality metrics were further assessed using VariantQC, including total processed loci, number of called loci, and filtering rates. Only variants marked as PASS were retained for downstream association analyses.

### 2.7 Ancestrality

The ngsRelate tool was used to detect individuals with a high degree of relatedness (14). Subsequently, the VCF file was converted to the standard PLINK format (v1.90b7.1) (15) and subjected to linkage disequilibrium pruning (indep-pairwise = 50 10 0.1), corresponding to a size of 50 SNPs, a step size of 10 SNPs, and an r^2^ threshold of 0.1. Admixture analysis and estimation of the number of populations were conducted using ADMIXTURE (v1.3.0) (16), considering K values between 2 and 5. The 1,000 Genomes Project data was used as a reference for ancestry analysis and was incorporated into the FRAPOSA algorithm (17) with default settings. Additionally, the Gencode.exome database and the EthSEQ tool (18) were used for principal component analysis (PCA) visualization and ancestry classification. To account for population stratification in our cohort, the PCAs from this analysis were subsequently included as covariates in logistic regression models.

### 2.8 Analysis with machine learning algorithms

After applying quality control filters to the VCF file, variants with more than 10% missing genotype calls were excluded. As only a small amount of sporadic missing data remained after this filtering step, missing genotypes were imputed using the most frequently observed genotype (mode) for each variant. This strategy was chosen because the study focused exclusively on variants directly identified by whole-exome sequencing, and the use of reference panel-based imputation methods could introduce bias in this highly admixed cohort. After this stage, the data were standardized and divided into training and test sets. The XGBoost was selected due to its more stable feature importance estimation, as Support Vector Machine showed a tendency to overemphasize specific features, potentially biasing the selection process. With XGBoost as the base estimator, the Recursive Feature Elimination (RFE) method was employed to reduce the number of dimensions and select the most significant variants (19). The ML framework was restricted to genomic features, and clinical covariates were intentionally excluded from the feature selection stage. This design choice was adopted because the primary objective of the analysis was to identify a potential host genetic signature associated with LC susceptibility. Inclusion of highly predictive clinical variables, such as age, acute disease severity, and comorbidities, could dominate feature importance rankings and obscure the contribution of genomic variants during recursive feature selection. Instead, clinical covariates were evaluated separately through descriptive association analyses and incorporated in downstream logistic regression validation models adjusted for age, sex, and ancestry principal components. This strategy allowed the ML framework to prioritize genomic signals while preserving independent statistical assessment of relevant clinical factors. Because of the analysis of exploratory nature, RFE was performed on the complete dataset, assessing different SNP quantities (from 1 to 50). The ROC AUC metric was used to see how well the XGBoost model worked. The SHAP (Shapley Additive Explanations) values were calculated exclusively on the held-out test set after model training. Because feature selection was performed within the same dataset, model performance metrics should be interpreted cautiously.

### 2.9 Statistical analysis

The chi-square test was used to analyze categorical variables, and a significance level of *p* < 0.05 was established. All analyses were conducted using Python 3.12.11. The following section presents our cohort’s results, with a focus on identifying genetic variants associated with LC. To provide an independent statistical assessment of the selected variants, logistic regression analyses were performed using PLINK v1.9 under an additive genetic model. Odds ratios (OR) and 95% confidence intervals (CI) were estimated. To minimize model instability given the sample size, regression analyses were restricted to variants prioritized by the ML framework. Analyses were adjusted for sex, age, and principal components of ancestry (PCA). *p*-values were corrected for multiple tests using the Benjamini-Hochberg false discovery rate (FDR) procedure. FDR correction was applied across all SNPs tested in the logistic regression association analysis. Variants with FDR-adjusted *p*-values < 0.05 were considered statistically significant.

### 2.10 Functional annotation of prioritized variants

To explore potential biological relevance of the prioritized variants, functional annotation was performed using the Combined Annotation Dependent Depletion (CADD, v1.6) scoring system. CADD PHRED-scaled scores were obtained for all selected variants to estimate their predicted deleteriousness. Given that most prioritized variants were intronic, this analysis aimed to assess potential regulatory relevance rather than protein-coding disruption. Additionally, publicly available expression quantitative trait loci (eQTL) data and Splicing quantitative trait loci (sQTL) from the (v8- GRCh38) database were consulted to investigate whether prioritized variants were associated with gene expression changes in relevant tissues. The GTEx portal was used solely to obtain eQTL and sQTL annotations for the prioritized variants, whereas the reported sequela frequencies were derived from our own clinical-epidemiological dataset. Variants were queried using rsID and genomic coordinates to ensure accurate matching. Only variants directly represented in the GTEx dataset were considered; linkage disequilibrium proxies were not systematically evaluated. Variants that do not present in the database were reported as not represented.

## 3 Results

### 3.1 Association of sociodemographic factors, comorbidities, and biomarkers with Long COVID development

Of the 312 sequenced individuals (Supplementary Table S2), 66.03% reported persistent symptoms following SARS-CoV-2 infection. Severity (FDR-adjusted *p* < 0.001), age group (FDR-adjusted *p* = 0.017), and comorbidities related to chronic pulmonary disease (FDR-adjusted *p* = 0.003), chronic cardiovascular disease (FDR-adjusted *p* = 0.015), Diabetes Mellitus (FDR-adjusted *p* = 0.005), and obesity (FDR-adjusted *p* = 0.002) were significantly associated with the persistence of sequelae. Female participants were predominant, representing 56.73% of our cohort. The most frequently reported persistent clinical manifestations included fatigue (35.26%), joint pain (26.28%), myalgia (16.99%), dyspnea (23.40%), and neurosensory alterations, such as loss of smell (17.95%), taste (16.03%), and dysautonomia (11.54%).

### 3.2 Ancestrality

**Figure 1:**
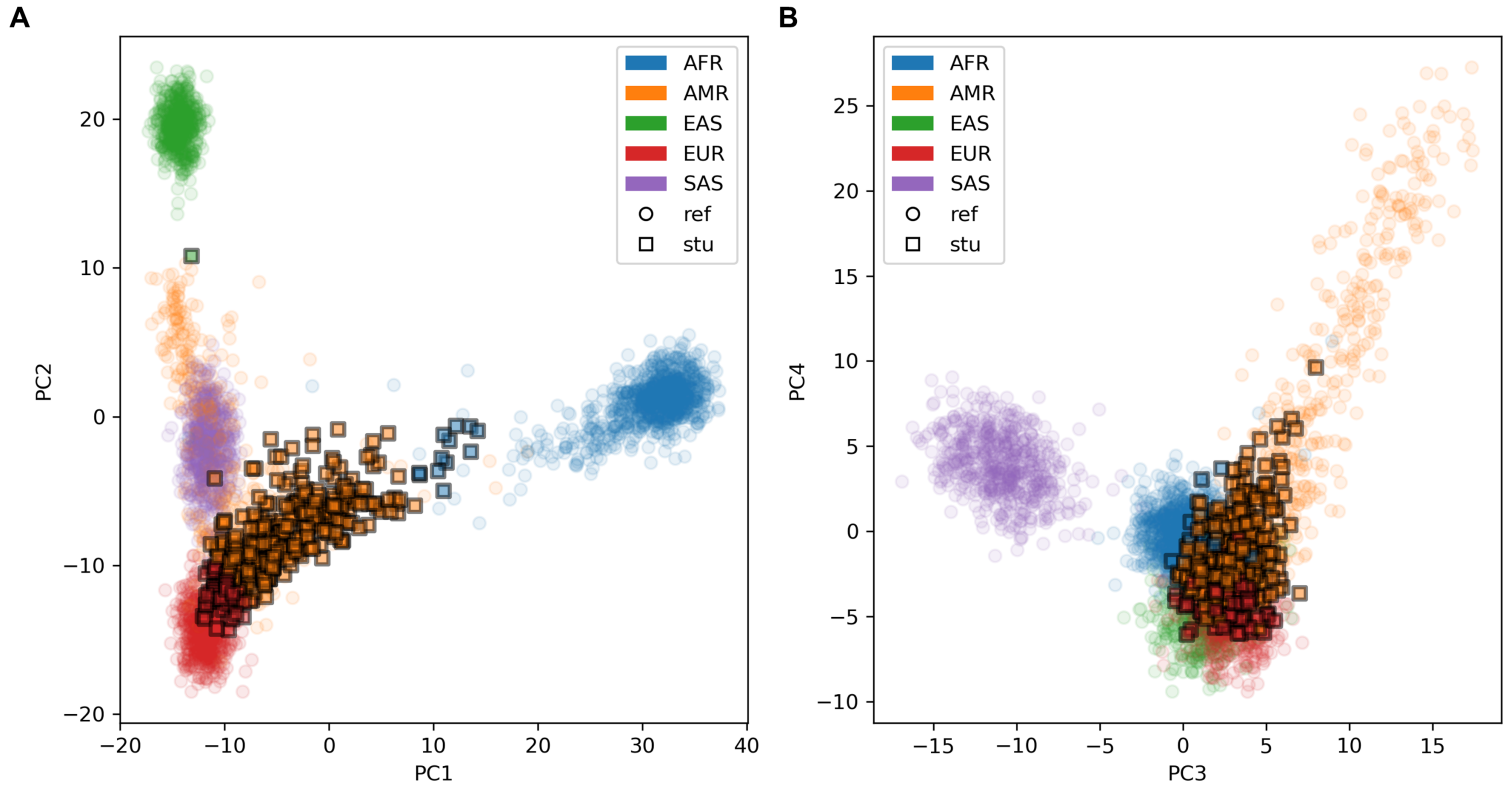
Ancestrality. **(A)** (PC1 and PC2) and **(B)** (PC3 and PC4). Colors represent different genomic populations: blue for African (AFR), orange for Amerindian (AMR), green for East Asian (EAS), red for European (EUR), and purple for South Asian (SAS). Circles indicate reference samples (ref), and squares represent the studied samples (stu).

The PCA analysis revealed that most of the sequenced samples clustered near the Amerindian (AMR) group, reflecting the characteristic admixture pattern of Latin American populations. A smaller proportion of samples clustered with the European (EUR), African (AFR), and East Asian (EAS) reference populations, highlighting the genetic heterogeneity within our cohort (Figure 1). We also investigated kinship among study participants to assess its influence on the results of the genetic association tests.

### 3.3 Genomic aspects

After sequencing, a total of 651,652 loci were identified through raw data analysis performed by the DRAGEN pipeline itself (Illumina). The vast majority of sequenced variants were classified as PASS (70.3%), indicating high-confidence calls. Among the filtered variants, 26.5% were annotated as MosaicLowAF, a proportion flagged in relation to quality and depth-related filters, including LowDP (0.24%). All variants that did not meet DRAGEN’s rigorous quality standards for SNPs or indels were represented by less than 0.1% of the calls. After this initial filtering, 192,876 variants remained, consisting of SNPs (97.9%), followed by deletions (0.9%) and insertions (0.6%). Complex and mixed variants were infrequent (<1%). Additional filters described in the methodology section reduced the dataset to approximately 8,000 SNPs. This reduction is a result of applying quality filters, frequency filters, linkage disequilibrium pruning (indep-pairwise = 50 10 0.1), and Hardy–Weinberg balance filtering to the controls.

### 3.4 Machine learning framework for host genomic variant prioritization

**Figure 2:**
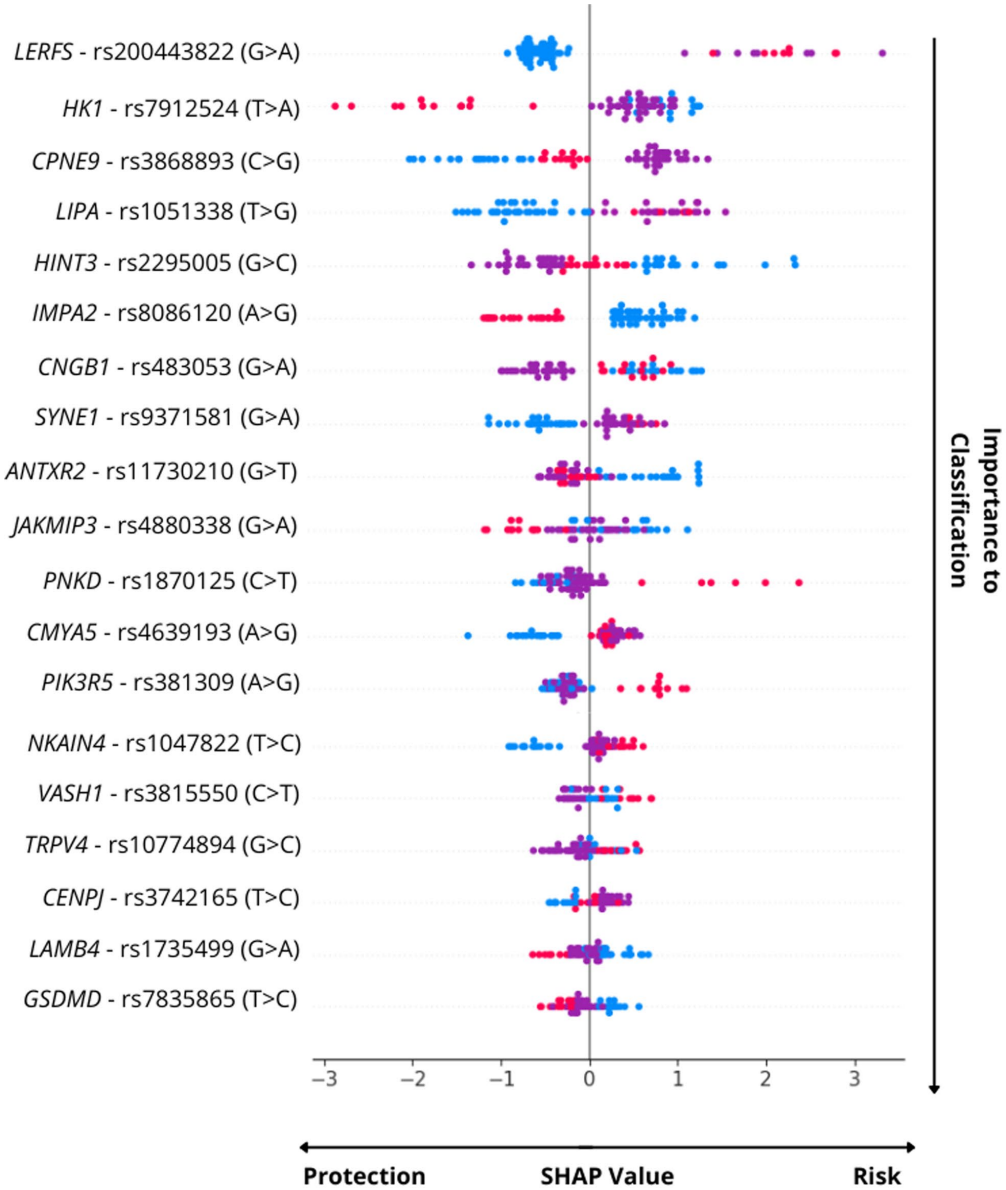
SHAP summary plot illustrating the contribution of each genotype to the machine-learning–based Long COVID classifier: influence of every chosen genotype (feature) on the results of ML models and genomic polymorphisms (LC or non-LC). Reference homozygotes are shown in blue, heterozygotes in purple, and alternative homozygotes in pink. The SHAP analysis that was done solely on the test dataset is shown in the figure.

Within the analyzed cohort, the classifier achieved an accuracy of 87%, an F1 score of 90%, and an area under the curve (AUC) of 92%, reflecting internal separation performance. In the SHAP plot (Figure 2), 11 SNPs showed positive contributions of the variant allele to the internal classification of LC cases within the model, including *PNKD* (rs1870125), *CMYA5* (rs4639193), *SYNE1* (rs9371581), *LERFS* (rs200443822), *LIPA* (rs1051338), *TRPV4* (rs10774894), *VASH1* (rs3815550), *CNGB1* (rs483053), *PIK3R5* (rs381309), *NKAIN4* (rs1047822), and *CENPJ* (rs3742165). Conversely, 8 SNPs displayed negative contributions of the variant allele within the model, including *CPNE9* (rs3868893), *ANTXR2* (rs11730210), *LAMB4* (rs1735499), *GSDMD* (rs7835865), *HK1* (rs7912524), *JAKMIP3* (rs4880338), *IMPA2* (rs8086120), and *HINT3* (rs2295005).

Importantly, the ML framework was employed as a feature prioritization strategy rather than a predictive modeling pipeline. Accordingly, the reported performance metrics reflect internal discriminative capacity within the analyzed cohort and should not be interpreted as measures of model generalizability or estimates of clinical prediction accuracy.

These initial findings motivated the subsequent analysis of how clinical factors modulate the impact of the identified variants, as discussed in the section below.

### 3.5 Logistic regression analysis of selected variants

Logistic regression analysis under an additive genetic model identified five variants that remained statistically significant after FDR correction. The variant at *LERFS* - rs200443822 was associated with increased odds of LC (OR = 6.21, 95% CI 2.98–12.91, FDR-adjusted *p* < 0.01). Similarly, the variant at *PNKD* - rs1870125 (OR = 2.33, 95% CI 1.52–3.57, FDR-adjusted *p* < 0.01) and *LIPA* - rs1051338 (OR = 2.87, 95% CI 1.77–4.65, FDR-adjusted *p* < 0.01) showed increased odds. In contrast, variants at *HK1* - rs7912524 and *LAMB4* - rs1735499 were associated with reduced odds of LC, with ORs ranging from 0.38 to 0.50 (all FDR-adjusted *p* < 0.01) (Table 1). The direction of effects was consistent between the ML contribution (SHAP directionality) and the odds ratios estimated by logistic regression.

**Table 1:** Logistic regression results for classifier-selected variants (additive model).

SNP	OR	L95	U95	*P* value	FDR*
*LERFS* - rs200443822 (G > A)	6.207	2.984	12.91	<0.01*	<0.01*
*HK1* - rs7912524 (T > A)	0.378	0.249	0.574	<0.01*	<0.01*
*LIPA* - rs1051338 (T > G)	2.871	1.771	4.653	<0.01*	<0.01*
*PNKD* - rs1870125 (C > T)	2.329	1.521	3.568	<0.01*	<0.01*
*LAMB4* - rs1735499 (G > A)	0.495	0.335	0.731	<0.01*	<0.01*
*NKAIN4* - rs1047822 (T > C)	1.526	1.059	2.198	0.02	0.07
*CPNE9* - rs3868893 (C > G)	1.522	1.036	2.235	0.03	0.07
*HINT3* - rs2295005 (G > C)	0.677	0.472	0.971	0.03	0.07
*SYNE1* - rs9371581 (G > A)	1.501	1.028	2.192	0.03	0.07
*GSDMD* - rs7835865 (T > C)	0.677	0.462	0.991	0.04	0.08
*JAKMIP3* - rs4880338 (G > A)	0.707	0.493	1.014	0.05	0.09
*ANTXR2* - rs11730210 (G > T)	0.717	0.501	1.027	0.06	0.10
*PIK3R5* - rs381309 (A > G)	1.401	0.972	2.018	0.07	0.10
*IMPA2* - rs8086120 (A > G)	0.702	0.459	1.073	0.10	0.13
*CENPJ* - rs3742165 (T > C)	1.227	0.845	1.781	0.28	0.34
*VASH1* - rs3815550 (C > T)	1.216	0.842	1.756	0.29	0.34
*CMYA5* - rs4639193 (A > G)	1.197	0.846	1.691	0.30	0.34
*TRPV4* - rs10774894 (G > C)	1.168	0.808	1.686	0.40	0.42
*CNGB1* - rs483053 (G > A)	0.884	0.617	1.269	0.50	0.50

Odds ratios (OR), 95% confidence intervals (L95–U95), nominal *p*-values, and false discovery rate (FDR)-adjusted *p*-values are shown for each SNP under an additive genetic model. OR >1 indicates increased odds of severe disease, whereas OR <1 indicates decreased odds within the analyzed cohort. Variants with FDR-adjusted *p*-values <0.05 were considered statistically significant. Associations should be interpreted in the context of multiple testing and the exploratory design of the study. The asterisk (*) indicates values that reached statistical significance.

### 3.6 *In silico* functional prediction using CADD

CADD v1.6 PHRED-scaled scores were obtained for all prioritized variants, including both intronic and exonic loci. Overall, most variants exhibited PHRED scores below 10, indicating the absence of strong predicted deleterious effects. One exonic variant in *LIPA* (rs1051338) reached a PHRED score of 10, suggesting moderate predicted functional relevance (Supplementary Table S4).

### 3.7 Regulatory annotation using GTEx v8 eQTL data

When the analysis was performed using GTEx v8 software, significant eQTL and sQTL signals were observed for the prioritized intronic variants in our study. In general, there was a predominance of association in neural and neuroendocrine tissues. For example, while *CNGB1* (rs483053) demonstrated regulatory effects in the hypothalamus, *PNKD* (rs1870125) and *NKAIN4* (rs1047822) were strongly associated with cortical regions and basal ganglia. Another gene that showed association was *GSDMD* (rs7835865), which presented extensive brain regulatory signals. The *HK1* variant (rs7912524) also showed notable expression variations in peripheral neural tissue. In cultured fibroblasts, there was a significant correlation with *ANTXR2* (rs11730210).

We also investigated exonic variants and their significant regulatory effects. We found that the variant in the *JAKMIP3* gene (rs4880338) and *CENPJ* (rs3742165) showed associations in several regions of the CNS, while *LIPA* (rs1051338) showed broad systemic and neuroendocrine regulation. The *VASH1* variant (rs3815550) was observed to be expressed in lung tissue, and *HINT3* (rs2295005) showed regulatory effects in mucosal and muscle tissues.

Not all prioritized variants were represented in the GTEx v8 dataset. However, all variants available for analysis showed statistically significant eQTL associations in at least one tissue. A complete summary of all significant tissue-specific results is provided in Supplementary Table S3.

### 3.8 Integrated genetic risk

Descriptive analysis of the selected variants indicated that the variants *NKAIN4* (rs1047822), *CMYA5* (rs4639193), *TRPV4* (rs10774894), and *PIK3R5* (rs381309) were among the most frequent within the selected set. Many carriers of these variants reported symptoms such as joint pain, fatigue, loss of taste or smell, and dysautonomia (Supplementary Figure S1). Variants in genes related to cytoskeletal and structural functions, including *SYNE1* and *VASH1*, were also frequently observed among individuals reporting autonomic and cognitive complaints. In contrast, *LERFS* (rs200443822) and *LIPA* (rs1051338) appeared less frequently in this comparison. These observations are descriptive and were not adjusted for multiple testing.

**Figure 3:**
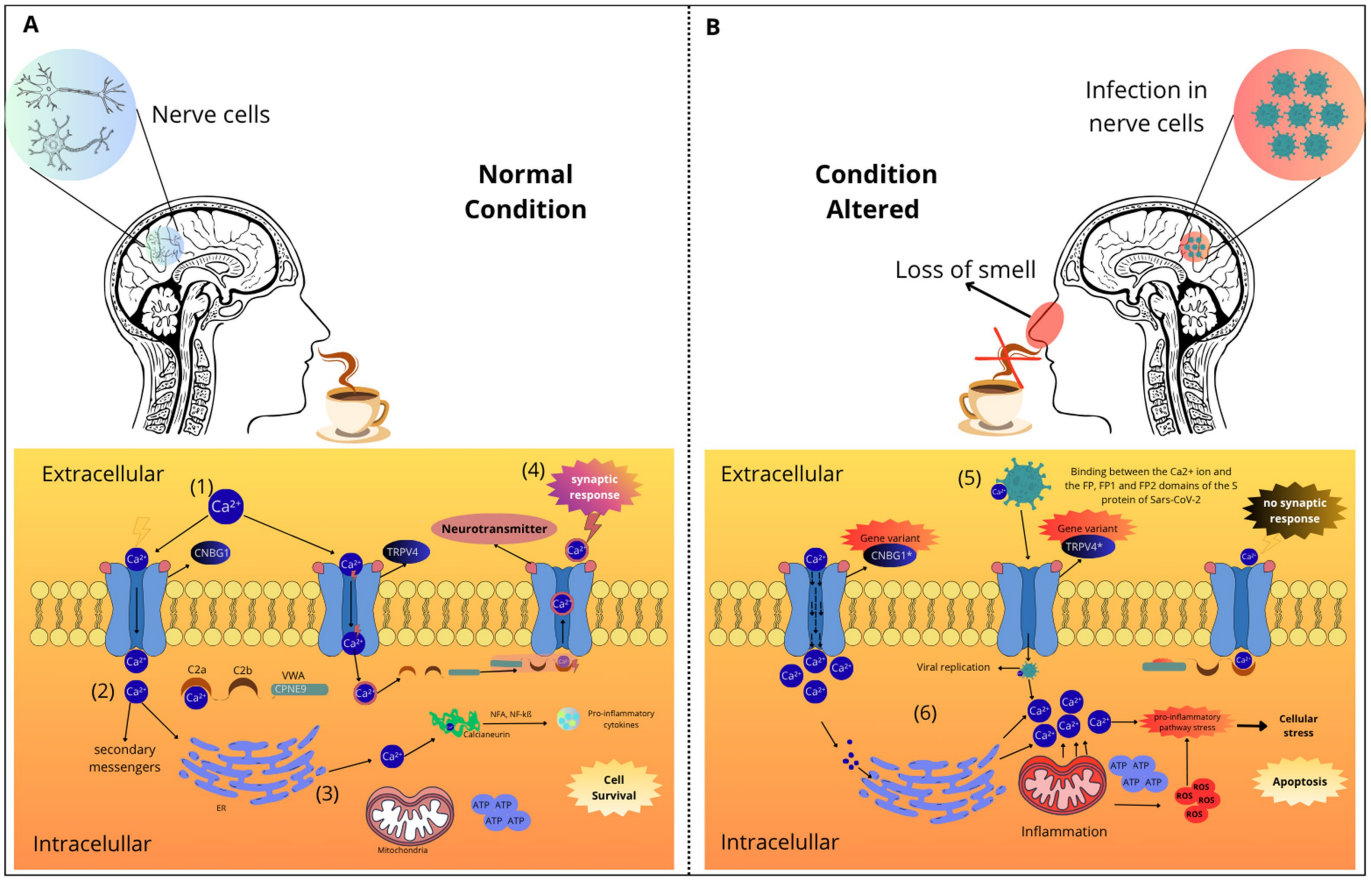
Conceptual model based on genetic associations and previously described calcium-dependent pathways. Panel **A** illustrates physiological conditions, showing healthy neuronal cells with preserved olfactory function, normal Ca^2+^ signaling, neurotransmitter release, and neuronal survival. Panel **B** depicts a hypothetical pathological scenario associated with viral infection, characterized by viral replication, altered Ca^2+^ signaling, cellular stress, inflammation, apoptosis, and impaired synaptic responses, highlighting the potential contribution of genetic variants to olfactory dysfunction. (1) The figure demonstrates that under physiological conditions, calcium ions can enter the intracellular space through ion channels such as the TRPV4 channel and the CNGB1 channel. (2) Subsequently, once these ions enter the cell, Ca^2+^ acts as a second messenger, interacting with intracellular receptors, including those located in the endoplasmic reticulum. (3) This interaction is interesting because it will contribute to subsequent signaling cascades. (4) Furthermore, these pathways may participate in inflammatory modulation, cellular adaptation, and neurotransmitter release. (5) In a possible modified regulatory context, for example, during systemic viral stress, alterations in Ca^2+^ channel-mediated dynamics may alter intracellular calcium balance. (6) Sustained intracellular Ca^2+^ dysregulation may induce endoplasmic reticulum stress, mitochondrial dysfunction, ATP depletion, reactive oxygen species production, activation of pro-inflammatory pathways, and apoptosis, ultimately contributing to neuronal dysfunction and persistent olfactory impairment in Long COVID. In this context, we hypothesize that sustained disturbances in Ca^2+^ homeostasis may contribute to synaptic dysfunction and altered neuronal responsiveness, and be a mechanism to investigate in Long COVID.

To contextualize these findings biologically, we present a schematic representation of calcium signaling pathways potentially related to genes identified in this exploratory analysis (Figure 3). Variants in *TRPV4*, *CNGB1*, and *CPNE9* are located in genes known to participate in ion channel regulation and synaptic signaling. While this diagram illustrates plausible mechanistic connections, it does not imply direct functional effects of the identified variants, which require experimental validation.

In addition to genetic associations, we seek to assess how these factors translate into functional and psychosocial differences among participants, reflected in their quality of life, and this will be discussed in the next section.

### 3.9 Impact on quality of life

The EURO-QOL-5 Dimension questionnaire (20) was used to evaluate the 312 participants’ quality of life (QoL). People with LC performed worse than those without LC in every domain. The two domains with the biggest differences were the anxiety/depression domain (65.84% experienced emotional distress ranging from mild to extreme) and the pain/discomfort domain (50.99% reported some degree of pain).

Two years after infection, the differences were also noticeable in terms of overall health perception: 18.41% of LC patients rated their health as poor or very poor, compared to just 2.86% of non-LC patients. On the other hand, 97.14% of participants who were not LC said they were in good, very good, or excellent general health.

**Figure 4:**
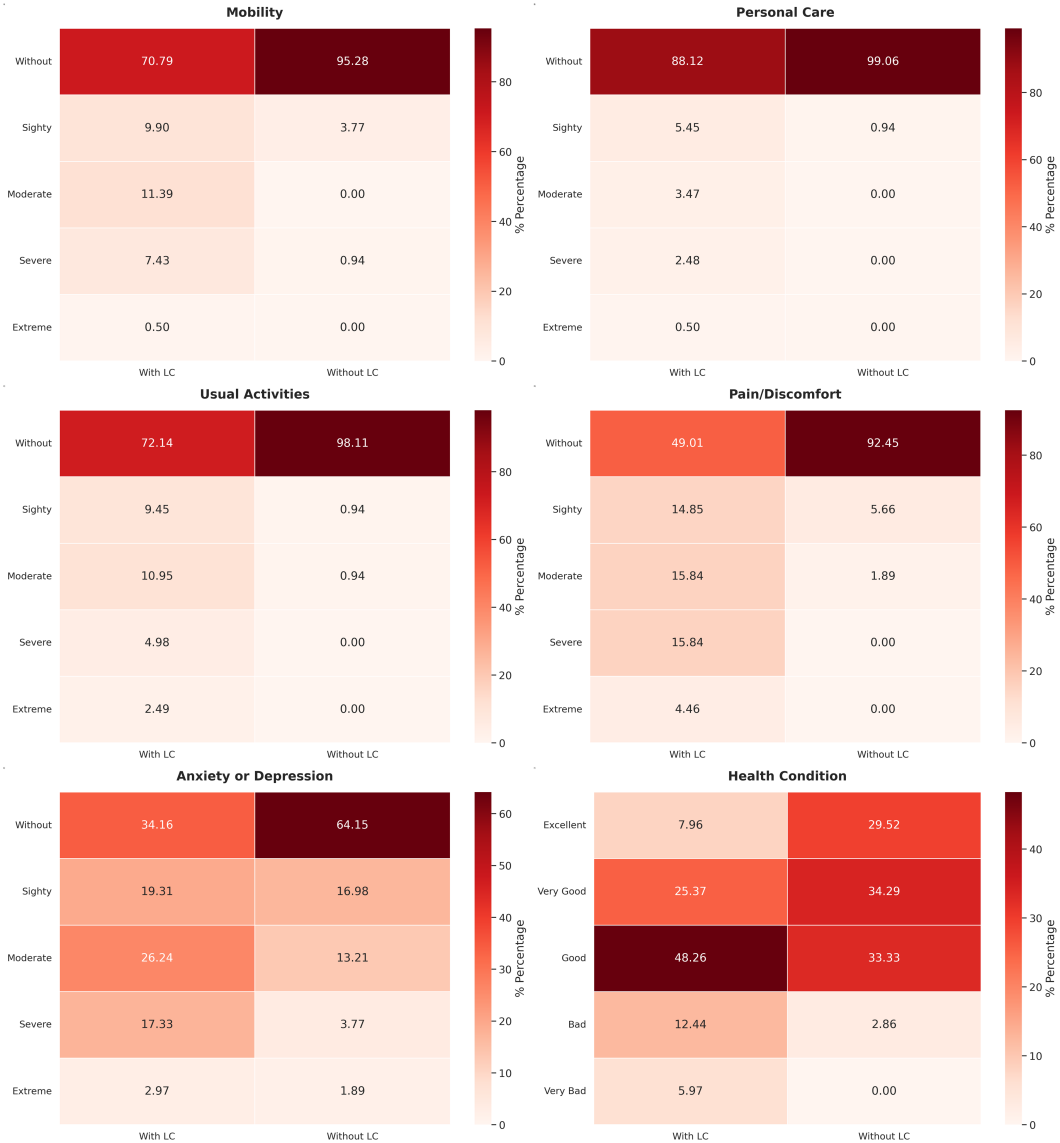
Heatmap. Heatmap on the percentage distribution of self-reported limitations across six domains of quality of life (mobility, self-care, usual activities, pain/discomfort, anxiety/depression, and general health) in individuals with and without LC.

These results, summarized in Figure 4, demonstrate the lasting impact of LC on quality of life, particularly in physical and mental well-being.

These results form the foundation for the following discussion of the clinical and genetic mechanisms underlying LC in our cohort.

## 4 Discussion

### 4.1 Association of sociodemographic factors and comorbidities with COVID severity

More severe forms of SARS-CoV-2 infection were linked to LC in our cohort, which is in line with earlier research showing that patients who needed intensive care were more likely to experience post-acute symptoms (10). Comorbidities like diabetes, obesity, and cardiovascular disease, as well as advanced age, were also closely linked to the condition. These factors, which are already known to influence the acute phase’s heightened inflammatory response (21, 22), also seem to have an impact on the sequelae that follows an infection.

Prolonged or dysregulated inflammation promotes long-term neurological outcomes and jeopardizes neuroimmune homeostasis (4). In turn, aging and comorbidities lead to endothelial dysfunction and chronic low-grade inflammation, which are linked to sustained glial activation and disruption of the blood–brain barrier (BBB) (23).

The variety of LC manifestations could be explained by the combination of these clinical characteristics and genetic predispositions. The identified variants were grouped into five functional axes for interpretation purposes: (i) calcium signaling and ion channels; (ii) bioenergetics and oxidative stress; (iii) neuroinflammation and the integrity of the blood–brain barrier; (iv) cytoskeletal organization and neuronal transport; and (v) lipidostasis and neuroinflammation. Furthermore, under an additive model, a number of variants continued to be linked to severity even after controlling for age, sex, and principal components of ancestry (Table 1).

### 4.2 Ion channel and Ca^2+^ signaling axis

According to Alves *et al*., calcium-dependent ion channels are essential for neurotransmission, synaptic plasticity, and neuronal survival (24). During viral infections, including SARS-CoV-2, Ca^2+^ homeostasis is disrupted, compromising cellular integrity and promoting neuroinflammation (25). Anosmia, cognitive impairments, and motor changes are among the chronic symptoms of LC that may be caused by variations in genes related to this pathway.

Among these, *TRPV4* stands out. Its sustained activation has been associated with neuroinflammation, glial dysregulation, and prolonged sensory deficits (26). In addition, it regulates olfactory epithelium regeneration by balancing neuronal proliferation and maturation (27). The rs10774894 variant, associated with inflammation, was found in over 70% of individuals in our cohort who presented with fatigue, loss of smell/taste, joint pain, and dysautonomia, suggesting a possible contribution to the sensory and inflammatory alterations observed in these individuals (Supplementary Figure S1).

In addition to being necessary for the transduction of olfactory and retinal signals, *CNGB1* also plays a role in Cyclic guanosine monophosphate (cGMP)-mediated synaptic plasticity (28, 29). In animal models, its absence has resulted in glial activation and neuronal degeneration (30). According to Lee *et al*., the *CNGB1* variant may impair channel stability, which would lead to the cognitive and sensory deficits associated with LC (31).

We observed that the rs483053 variant, located in *CNGB1*, showed a high frequency among patients who developed sequelae such as hypersensitivity to light/noise (75.6%), loss or reduction of smell/taste (73.2%/70.0%), migraine (74.6%), among other sensory or cognitive manifestations. Curiously, according to GTEx v8, this locus has been annotated as a significant sQTL in the hypothalamus. Given the role of the hypothalamus in homeostatic regulation and stress adaptation, subtle alterations in channel-mediated signaling could contribute to persistent dysregulation following systemic infection.

*CPNE9* is a calcium-dependent protein that is expressed in excitatory neurons and serves as a synaptic remodeling sensor (32, 33). Neurodegenerative disorders have been associated with changes in this pathway (34). The variant rs3868893 (*CPNE9*) was prioritized in our analysis; however, it was not available as an eQTL in the GTEx v8 portal. Therefore, the functional interpretation of this locus remains speculative and will require independent regulatory annotation and replication.

When viewed together, the mechanistic model (Figure 3) and the genetic findings (Figure 2) seem to point toward a possible convergence of *TRPV4*, *CNGB1*, and *CPNE9* within Ca^2+^-dependent signaling pathways. Rather than acting in isolation, these variants may reflect subtle shifts within a shared regulatory landscape. In the context of LC, where sensory and cognitive symptoms such as anosmia and mental fatigue are common, it is tempting to consider whether altered calcium signaling could represent one of several interacting mechanisms. While this interpretation remains exploratory, it offers a coherent biological framework that merits further investigation.

The PNKD protein is involved in the regulation of synaptic vesicle exocytosis and calcium channel-dependent neurotransmitter release (35, 36). Notably, in our cohort, the *PNKD* variant - rs1870125 remained statistically associated after correction for FDR in the regression analysis within this cohort (Table 1). Concomitantly, when the prioritized variant rs1870125 was queried in GTEx, it was identified as a strong eQTL in different brain regions.

Although these results are compatible with a possible mechanism involving calcium-dependent synaptic pathways in the development of LC, the consistency of the variant signal in different brain regions should be interpreted carefully.

The variants discussed in this section highlight the importance of proper neuronal excitability function and correct intracellular calcium dynamics. We emphasize that calcium homeostasis is fundamental for synaptic transmission and inflammatory signaling, and dysregulation of these pathways has been associated with neuroinflammatory processes.

### 4.3 Bioenergetics and oxidative stress axis

The mechanisms involved in oxidative stress and the maintenance of metabolic balance are essential for neural bioenergetics. Alterations in these pathways weaken neuronal resilience and may contribute to the persistent manifestations of LC. Within this axis, two genes stood out: *HK1* and *IMPA2*.

Hexokinase type 1 is encoded by HK1 and phosphorylates glucose initially during glycolysis (37). It inhibits apoptosis and reduces Reactive oxygen species (ROS) levels, in addition to maintaining Adenosine Triphosphate (ATP) synthesis (38, 39). Its consistent expression in neurons is essential to preserve brain plasticity (40). Beyond its function in reducing ROS, aberrant regulation of HK1 has already been associated with neurodegenerative diseases, COVID-19, and mitochondrial dysfunction under hypoxic conditions (40, 41).

Studies in animals have shown a transient reduction in HK1 expression in the hippocampus after trauma, followed by subsequent recovery (42). Furthermore, variants of this gene appear to provide greater energy stability, and in our cohort, the *HK1* variant (rs791252) remained significantly associated with LC, even after FDR correction, with a lower probability of CL manifestations. This association may suggest that the preservation of glycolytic efficiency and redox balance may modulate post-infectious vulnerability in our cohort.

Concurrently, inositol monophosphatase 2, an enzyme necessary for preserving sufficient myo-inositol (MI) levels in the central nervous system, is encoded by *IMPA2* (43). Psychiatric and neurological disorders have been associated with changes in MI levels (44). Anterior cingulate cortex MI levels have been found to be lower in post-COVID-19 patients (45). This imbalance may be exacerbated by variations in *IMPA2*, which would lead to neuropsychiatric symptoms.

Genes involved in mitochondrial bioenergetics and oxidative stress pathways may contribute to persistent fatigue and cognitive dysfunction observed in LC. Mitochondrial dysfunction has been described in chronic inflammatory states and may represent a shared vulnerability mechanism. The variants identified in this axis warrant further investigation to clarify potential regulatory effects.

### 4.4 Neuroinflammatory and blood–brain barrier axis

According to Elsheikh *et al*., ANTXR2 is a protein linked to neurodegenerative diseases and chronic inflammatory states (46). It also exhibits elevated expressions in hypoxic environments (47), which is consistent with post-viral alterations. According to GTEx v8, the intron variant rs11730210 (G > T) acts as a strong eQTL for *ANTXR2* in cultured fibroblasts (*p* = 5.1 × 10^−56^), with the T allele associated with reduced gene expression in a dose-dependent manner. These data indicate a regulatory effect at the transcriptional level in this cellular context. Although fibroblasts represent a peripheral tissue model, this finding suggests that genetic variation at this locus may influence *ANTXR2* expression under specific biological conditions. The broader physiological relevance of this regulatory effect remains to be established.

In models of brain injury, *PIK3R5*, a regulatory subunit of phosphoinositide 3-kinase *γ* (PI3Kγ), has demonstrated neuronal induction, aggravating cellular stress and neuronal death (48). Conversely, its selective inhibition reduces harmful neuroimmune reactions (49). These results raise the possibility that PI3Kγ signaling plays a role in the persistence of glial activation, which supports adaptive metabolic alterations that maintain long-term inflammatory reactions. Increased glycolysis, energetic support for reactive glial cells, and inflammation in LC are some of these alterations.

Another conceivable connection between systemic inflammation and neurological after effects is *GSDMD*, which causes pyroptosis and the release of Interleukin-1 Beta and Interleukin-18. *GSDMD* deletion preserved synaptic integrity and prevented hippocampus damage in animal models (50, 51). Furthermore, the intron variant (rs7835865) was identified as a significant eQTL in basal ganglia structures (caudate and putamen) according to GTEx v8, with the C allele associated with increased gene expression. Given that multiple sclerosis, Parkinson’s disease, and Alzheimer’s disease have already been linked to NLRP3–GSDMD inflammasome activation (52). This raises the possibility that synaptic dysfunction in LC may involve comparable mechanisms.

Finally, alterations in *LAMB4*, a laminin critical for the integrity of the BBB, may worsen neuroinflammatory dysfunctions in post-COVID-19 contexts. Its interaction with integrins and dystroglycans regulates BBB permeability and leukocyte migration (53), while receptors like laminin 37/67 kDa have been linked to degenerative and infectious diseases (54). Disturbances in the LAMB4–BBB axis may favor both viral entry and pathological remodeling of the extracellular matrix, contributing to prolonged neurological manifestations.

*LAMB4* remained significantly associated after FDR correction, with decreased odds observed in carriers, supporting a potential structural resilience component related to blood–brain barrier integrity within this cohort. Although functional validation is necessary, variants like rs1735499 (G > A), found within this exploratory ML framework, may represent genomic contexts possibly linked to pathways involved in BBB maintenance and neuroinflammatory responses.

### 4.5 Cytoskeletal organization and neuronal transport axis

Cognitive-motor function and synaptic connectivity depend on the neuronal cytoskeleton. Changes in this pathway may cause the cerebral network to deteriorate and prolong LC symptoms (55).

The regulator of microtubule dynamics (*CENPJ*) maintains cortical migration and neuronal localization. Dysfunctions in this gene have been associated with neuronal loss (56) and some of its variants can weaken the neuronal network and cause lasting cognitive deficits (57). In our cohort, although the rs3742165 variant showed a positive contribution to the model, it lost significance after statistical adjustments. However, GTEx analysis revealed significant eQTL signals in the hypothalamus and anterior cingulate cortex (Supplementary Table S3). Even though a direct mechanistic link cannot be established, they raise the possibility that microtubule instability may affect cognitive processes and interfere with neuroendocrine regulation.

In a complementary way, the VASH1–SVBP complex regulates *α*-tubulin detyrosination, a process essential for intracellular transport. Pathogenic variants in this axis have been associated with Alzheimer’s disease and other cognitive deficits (58). In the context of LC, chronic inflammation and oxidative stress can impair the tyrosination/detyrosination cycle, making microtubules less stable and directly impacting synaptic connectivity (59).

By interacting with Extracellular signal-related kinases 1 and 2 (ERK1/2)-dependent pathways, *JAKMIP3* (also known as *NECC2*) has been described as a possible link between intracellular signaling and cytoskeletal organization. In COVID-19, disruptions in axonal and synaptic transport have been increasingly described. Additionally, alterations in these signaling–cytoskeletal coupling mechanisms may contribute to post-infectious manifestations (60).

This exonic variant *JAKMIP3* rs4880338 (G > A) has been significantly associated with eQTL in several CNS regions, including the cerebral cortex, hippocampus, substantia nigra, hypothalamus, and pituitary gland. These regions are functionally relevant to cognitive and neuroendocrine regulation. Furthermore, In a post-viral context, where inflammatory stress, metabolic imbalance, and synaptic strain may coexist, even modest regulatory shifts might contribute to sustained neuronal dysfunction. However, although these signals suggest that this locus may be active in brain tissue, the biological implications of this variant still need to be clarified.

Lastly, the Na+/K + -ATPase interacts with NKAIN4 protein, which is highly expressed in the cortex and cerebellum. The integration of oxidative and inflammatory signals as well as neuronal excitability depends on this interaction. Prolonged activation of this pathway may encourage synaptic loss and progressive degeneration (61). More than 70% of the people in our group who had these gene variations displayed symptoms like joint pain, fatigue, dysautonomia, and loss of taste or smell (Supplementary Figure S1). These symptoms are consistent with sensory and autonomic neural circuit dysfunctions reported in earlier research.

Interestingly, in addition to the high frequency of this variant in sequelae such as fatigue and loss of smell/taste, although these findings do not establish causality, we observed a wide distribution of this eQTL signal in limbic, basal ganglia, cortical, and neuroendocrine structures. Thus, this distribution suggests that allelic variation in this locus may exert a broad modulatory effect on neural gene expression (Supplementary Table S3).

When taken together, variations in *CENPJ*, *VASH1*, *JAKMIP3*, and *NKAIN4* suggest that the compromised cerebral architecture and the distinctive cognitive and motor deficits of LC may be linked to cytoskeletal dysregulation in conjunction with the post-infection inflammatory environment.

### 4.6 Lipidostasis–neuroinflammation axis

In this exploratory ML framework, the variant rs1051338 (T > G) in *LIPA* remained significantly associated with LC after FDR correction, with increased odds observed among carriers. Furthermore, GTEx interrogation revealed eQTL signals across systemic and neuroendocrine tissues, including whole blood (*p* = 5.53 × 10^−71^), lung (*p* = 1.75 × 10^−28^), adrenal gland (*p* = 1.02 × 10^−20^), and pituitary (*p* = 4.42 × 10^−7^), following a consistent additive expression pattern (TT > TG > GG). The moderate CADD PHRED score of 10 supports a regulatory rather than highly disruptive coding effect.

The degree of infection and its enduring symptoms have been found to be influenced by the lipidostasis–neuroinflammation axis. The intracellular buildup of lipid droplets and the inflammatory activation of microglia are controlled by the *LIPA* gene, which codes for the lysosomal acid lipase (LAL) that breaks down lipids lysosomally (62, 63).

In experimental models, lipid droplet accumulation, macrophage infiltration, and intensified inflammatory phenotypes are the outcomes of LAL deficiency (63). The brain is also affected by the interface between innate immunity and lipid metabolism that has been previously identified in the intestine and other peripheral tissues. Functional changes in LIPA in this area encourage lipid buildup in microglia, resulting in a pro-inflammatory phenotype akin to that seen in aging or hypoxic/ischemic brains (64).

Moreover, transcriptomic evidence has shown that increased expression of *LIPA* in activated microglia in neurodegenerative diseases like Alzheimer’s is associated with the control of lipid autophagy and the modification of the inflammatory response (65). This axis is biologically plausible given the known interplay between lipid metabolism and epigenetic regulation. Metabolic intermediates such as acetyl-CoA and S-adenosylmethionine serve as cofactors in histone modifications and may influence inflammatory gene expression in contexts of metabolic stress (66). These observations suggest that lysosomal lipid metabolism could represent a biologically plausible pathway contributing to chronic neuroinflammatory signaling in LC, warranting further investigation.

### 4.7 Underexplored genes

In this investigation, we identified genes that have been little explored in the context of COVID-19 but that may be involved in structural, metabolic, and inflammatory mechanisms. *SYNE1*, a regulator of nuclear organization, has been reported as differentially expressed in SARS-CoV-2 infection models (67).

Conversely, *LERFS*, a lncRNA linked to the mechanistic target of rapamycin complex 1 pathway, may modulate cellular reactivity in settings of sustained inflammation, as observed in rheumatoid arthritis (68). The clinical spectrum of acute infection was significantly correlated with the rs200443822 variant in *LERFS* in our cohort (FDR = 0.030), indicating that dysregulation of this pathway in the early stages of the disease may be linked to a propensity for persistent LC manifestations. While we do not fully understand the regulatory function of the rs200443822 variant, its persistence even after statistical adjustment suggests the possible involvement of additional regulatory mechanisms, or more specific mechanisms, at play. Further functional studies will be essential to clarify its biological role.

The *CMYA5* gene, which is highly expressed in cardiac tissue, may be associated with secondary processes of viral myocarditis (69). Although its variant appeared in more than 70% of mutated individuals presenting with symptoms such as fatigue, loss of smell/taste, joint pain, and dysautonomia (Supplementary Figure S1), there is limited information available in the literature.

Potential modulatory activity in intracellular cascades sensitive to metabolic status is demonstrated by gene *HINT3* (70). Despite not being among the conventional indicators of viral response, these genes are at the intersection of oxidative stress, nuclear reorganization, and extracellular matrix dysfunction, indicating new avenues for understanding the cellular mechanisms underlying LC.

Given the exploratory nature of pathway grouping and the predominance of non-coding variants, these mechanistic interpretations should be considered hypothesis-generating.

### 4.8 Translational implications

These observations should be considered as hypothesis-generating. Although the analysis suggests that host genomic variation may contribute to clinical heterogeneity, it is not possible to infer, from the current dataset, any predictive or clinical utility, even though the results have demonstrated biological cohesion. Therefore, any application with the potential for risk stratification or possible discoveries of clinical biomarkers requires independent replication, as well as calibration assessment and prospective validation.

### 4.9 Quality of life

The study’s findings demonstrate the extensive and long-lasting declines in quality of life that occur after contracting SARS-CoV-2. These results are in line with research showing a persistent drop in both mental and physical health indices (71, 72). In addition to the direct symptoms of the infection, social isolation, unstable economic conditions, and decreased physical activity have also contributed to this decline, aggravating both physical and psychological symptoms (73, 74). A persistent cycle of vulnerability results, necessitating public health attention that goes beyond the clinical treatment of after effects.

### 4.10 Limitations

In interpreting our findings, some limitations of this study should be noted. Firstly, the statistical power to detect small-scale effects is still low, even with a sample size considered adequate. In this context, variants prioritized through exploratory and data-driven approaches may be susceptible to inflated effect size estimates. Therefore, the odds ratios reported in this study should, particularly for *LERFS* rs200443822 (OR = 6.21), be interpreted cautiously, as their magnitude may be overestimated in the discovery cohort. Replication in larger and independent populations will be necessary to validate these associations and obtain more precise effect size estimates. Thus, the present findings should be considered hypothesis-generating rather than definitive evidence of causality.

Secondly, since the participants are mainly from a mixed-race Brazilian group with Amerindian predominance, the results presented here may not be generalizable to other population contexts. On the other hand, this characteristic is also quite advantageous, since we are contributing something new to the literature by presenting a distinct and underrepresented group in global genomic studies.

Thirdly, this study did not incorporate explicit analyses of genetic ancestry in the ML model to assess whether population structure could have influenced the results. However, we performed a subsequent analysis with traditional logistic regression models implemented in tools such as PLINK, which allowed the inclusion of principal components as well as important covariates. We believe it is important that future studies incorporate principal components into their studies to refine model calibration and increase generalization to diverse populations.

Another limitation we acknowledge is the lack of longitudinal data, which prevents a complete understanding of the estimated variation in LC over time and how it may dynamically react to genetic differences. Simultaneously, we reinforce here the need for replication in independent populations, as well as the use of longitudinal designs that could provide us with more precise information about LC and its genetic etiology.

## 5 Conclusion

In conclusion, our study aimed at an exploratory analysis that assessed host genomic variation in relation to LC in a mixed-race Brazilian cohort using techniques such as whole-exome sequencing and ML. Through supervised prioritization of genetic variants, followed by logistic regression adjusted for age, sex, and principal components of ancestry, we obtained a subset of candidate variants associated with long-term COVID-19 in this cohort.

The prioritized loci included intronic and exonic variants, which were distributed across pathways primarily related to immune regulation, mitochondrial metabolism, neuroinflammatory signaling, and structural integrity. Although several variants demonstrated regulatory signals in tissue-specific expression datasets, these findings cannot be interpreted as evidence of directly functional effects.

We understand that rather than indicating single-gene causality, the associations observed here may reflect context-dependent modulation of interconnected biological systems. This is mainly due to the exploratory nature of the study, the absence of external replication, and the lack of experimental validation. Therefore, these findings should be regarded as hypothesis-generating and independent cohorts, and functional studies will be needed to determine the reproducibility and biological relevance of these observations.

## Data Availability

The datasets generated and/or analyzed during the current study are not publicly available at this time because they are being used in ongoing analyses related to the broader research project. Data will be made available from the corresponding author upon reasonable request and following completion of these analyses, subject to institutional and ethical restrictions regarding participant privacy and confidentiality.

## Glossary

ANTXR2: ANTXR cell adhesion molecule 2
CENPJ: Centromere protein J
CMYA5: Cardiomyopathy associated 5
CNGB1: Cyclic nucleotide gated channel subunit beta 1
CPNE9: Copine family member 9
GSDMD: Gasdermin D
HINT3: Histidine triad nucleotide binding protein 3
HK1: Hexokinase 1
IMPA2: Inositol monophosphatase 2
JAKMIP3: Janus kinase and microtubule interacting protein 3
LAMB4: Laminin subunit beta 4
LERFS: Fibroblast-like synoviocyte migration, SYNCRIP interacting
LIPA: Lipase A, lysosomal acid type
NKAIN4: Sodium/potassium transporting ATPase interacting 4
NLRP3: NOD-like receptor protein 3
PIK3R5: Phosphoinositide-3-kinase regulatory subunit 5
PNKD: PNKD metallo-beta-lactamase domain containing
SYNE1: Spectrin repeat containing nuclear envelope protein 1
TRPV4: Transient receptor potential cation channel subfamily V member 4
SVBP: Small Vasohibin Binding Protein
VASH1: Vasohibin 1
